# A whole-body mechanistic physiologically-based pharmacokinetic modeling of intravenous iron

**DOI:** 10.1007/s13346-024-01675-x

**Published:** 2024-07-24

**Authors:** Xiaoqing Fan, Kangna Cao, Raymond S. M. Wong, Xiaoyu Yan

**Affiliations:** 1 School of Pharmacy, Faculty of Medicine, The Chinese University of Hong Kong, Shatin, 8Th Floor, Lo Kwee-Seong Integrated Biomedical Sciences Building, Area 39, Hong Kong SAR, China; 2 Division of Hematology, Department of Medicine and Therapeutics, Faculty of Medicine, The Chinese University of Hong Kong, Hong Kong SAR, China

**Keywords:** PBPK modelling, Iron, Ferric carboxymaltose, Interspecies scaling

## Abstract

Iron is essential for every cell of the mammalian organism. Iron deficiency is a major public health issue worldwide. Intravenous (IV) iron therapy has been used to treat anemia. However, IV iron therapy is known far away from ideal because the quantitative relationship between the pharmacokinetics and biodistribution of IV iron under different iron statuses remains unclear. Patients are known to suffer adverse effects from excessive iron accumulation. Our objective was to develop a physiologically based pharmacokinetic (PBPK) model of iron in mice and validate its application for predicting iron disposition in rats and humans. Previously published data on iron were collected for constructing the PBPK model of iron in mice, and then extrapolated to rats and humans based on physiologically and chemically specific parameters relevant to each species. The PBPK model characterized the distribution of iron in mice successfully. The model based on extrapolation to rats accurately simulated the ferric carboxymaltose (FCM) PK profiles in rat tissues. Similarly, the observed and simulated serum PK of FCM in humans were in reasonable agreement. This mechanistic whole-body PBPK model is useful for understanding and predicting iron effects on different species. It also establishes a foundation for future research that incorporates iron kinetics and biodistribution, along with related clinical experiments. This approach could lead to the development of effective and personalized iron deficiency anemia treatments.

**Graphical Abstract:**

Major components and processes of whole-body systemic iron trafficking.

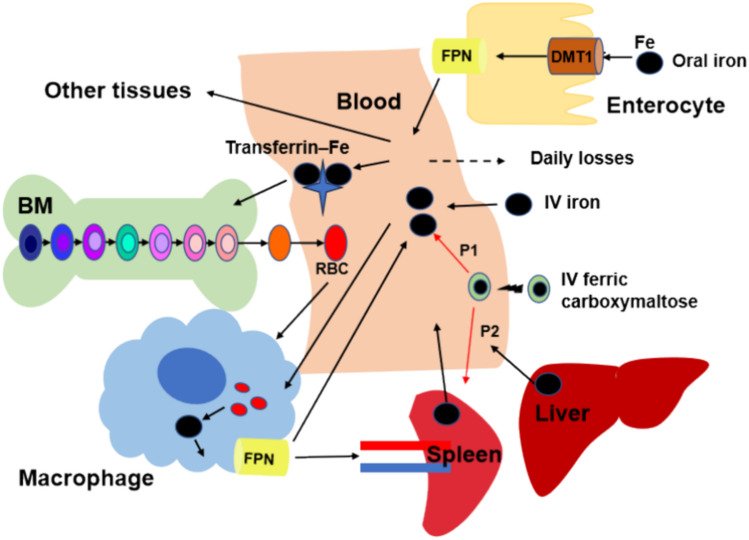

**Supplementary Information:**

The online version contains supplementary material available at 10.1007/s13346-024-01675-x.

## Introduction

Iron is a vital trace element required for a variety of vital physiological functions in humans and other mammals due to its ability to accept and lose electrons. It is used for oxygen transport, DNA metabolism, cellular energy generation, and also for cell proliferation and differentiation [1]. Deregulated iron metabolism, which results in iron deficiency (having too little) or iron overload (having too much), is associated with many different diseases. For example, excesses of iron can produce reactive oxygen species (ROS) that damage lipid and DNA, causing cell death through ferroptosis [2, 3]. On the contrary, iron deficiency significantly contributes to the pathogenesis of anemia in chronic kidney disease (CKD) [4]. Thus, treating iron deficiency is essential for managing anemia, especially in patients who necessitate kidney replacement therapy [5]. Iron deficiency anemia (IDA) is the most prevalent nutritional deficiency worldwide, affecting approximately 30% of the population [6].

**Fig. 1:**
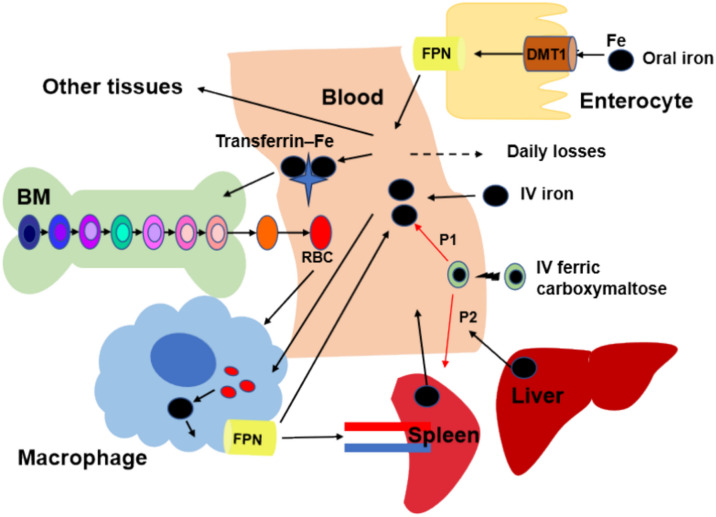
Major components and processes of whole-body systemic iron trafficking. Oral iron is absorbed across the enterocytes, while IV iron enters the plasma directly. Iron binds to transferrin in the plasma, and then be transported and taken up by the bone marrow (BM) for red blood cells (RBC) production or the liver or spleen for storage. Iron is recycled when macrophages take up senescent RBCs and release iron back to the plasma pool

Given iron imbalance results in various pathological conditions, mammals have developed complex mechanisms for controlling whole-body iron concentrations within normal physiological levels to maintain the normal structure and function of the organs [7]. To maintain iron homeostasis, various iron regulatory proteins coordinate with multi-organ systems throughout the entire transportation and storage of iron (Fig. 1) [8, 9]. Iron binds to plasma transferrin for distribution to tissues. The majority of iron is utilized in the bone for the synthesis of hemoglobin (Hgb) in red blood cells (RBCs). Senescent erythrocytes are phagocytosed by macrophages, releasing iron from catabolized Hgb back into the circulation [9].

Erythropoiesis (A process of producing RBCs) is the major iron consumer in the body because iron is a vital constituent of Hgb, and this need is satisfied by maintaining a sensitive regulation of iron levels [10]. Iron deficiency frequently leads to anemia, especially in CKD. The 2012 KDIGO guidelines advocate for the use of erythropoiesis-stimulating agents (ESAs, e.g. erythropoietin), iron, and transfusions in the treatment of anemia among CKD patients [11]. Over the past decade, practice patterns have shifted towards decreased use of ESAs and increased use of iron supplementation in many countries, due to adverse outcomes associated with ESAs [12]. Multiple oral and intravenous (IV) iron agents have been approved [12]. Recent randomized clinical trials (RCTs) have demonstrated the superior efficacy and similar safety of IV iron preparation compared with oral iron preparations, for the management of anemia in CKD. However, both oral and IV iron formulations are recognized as suboptimal, primarily due to concerns regarding tolerability and safety [13].

The diagnosis of iron deficiency and iron treatment in anemic CKD traditionally relies on serum transferrin saturation (TSAT), reflecting circulating iron levels, and serum ferritin, indicating iron stores. In CKD, absolute iron deficiency is characterized by relatively low TSAT and ferritin, while functional iron deficiency is marked by relatively low TSAT and normal ferritin levels. However, RCTs suggest that these biomarkers for iron deficiency in CKD lack reliability in evaluating body iron stores, predicting the response to iron therapy, and guiding iron dosing regimens. Consequently, the dose of IV iron preparation is not optimal [14]. Optimization of IV iron agent dosage has been recognized as a critical research priority in anemia management for CKD patients^1^. Given the complexity of the PK behavior of IV iron agents, understanding the iron disposition in various tissues (including plasma) post-treatment is crucial for optimizing the dose.

Physiologically based pharmacokinetic (PBPK) modeling is a powerful tool that combines the mathematical description of physiological and biological processes to assess the disposition of a xenobiotic in tissues^14^. Compared with traditional compartmental modeling, PBPK modeling is a more mechanistic method that treats the body as interconnected physiological compartments linked by blood flow, facilitating extrapolation across species [15, 16]. Therefore, PBPK models can be applied to predict xenobiotic exposure in various human organs or tissues based on that in animals. Moreover, it employs mass balances within the body, thus allowing for predicting the time-course profiles of toxicant amounts in tissues and fluids, helping to deepen the comprehension of therapeutical benefits and adverse effects, and eventually contributing to optimal dosing regimens [17].

There are other efforts in the literature for developing mathematical models for iron agents [18–21]. However, comprehensive whole-body PBPK models for iron are currently absent from the literature. Developing a whole-body PBPK model for iron would be beneficial, offering deeper insights into the disposition of iron in vivo and prospective applications in optimizing iron therapy [22]. Here, we constructed a whole-body mechanistic PBPK model that can predict iron tissue distributions using digitized published data for iron in mice [18, 23], and further extrapolated the mouse PBPK model to rats and humans to assess its application in predicting the tissue disposition of iron across different species. Establishing a quantitative relationship between the dose and PK of an IV iron paves the way for a more precise and scientific method to optimize IV iron dosages.

## Methods

### Software

The PBPK model was established using NONMEM (Version 7.5, Icon plc, USA); Model diagnostics utilized the R program (version 4.1.1, www.r-project.org) for analysis. Graphical visualization was created using the R program. Published tissue concentration–time curves of iron were digitized using the WebPlotDigitizer (version 4.5, https://apps.automeris.io/wpd/).

## Data collection

The PK and biodistribution studies of iron in mice [18, 23], rats [24], and humans [25] were taken from the published literature, which was utilized to build and verify the PBPK model. Because the PK study of iron in mice provided the most sufficient data on iron disposition in multiple tissues under different iron statuses, serving as the basis for developing the mouse PBPK model [23]. Briefly, iron-deficiency, iron-adequate, and iron-loaded mice were induced by feeding mice with a diet that contained different iron content. The mice were intravenously injected with 0.2 μmol ^59^Fe/kg. The mice were killed and dissected 15 min, 12 h, 24 h, and days 4, 7, 14, and 28 post IV ^59^Fe -administration to measure ^59^Fe -activity in organs.

Iron concentration–time data after administration of the IV iron preparation ferric carboxymaltose (FCM) in rat tissues [24] and serum PK profile in humans [25] were utilized to evaluate the interspecies scaling of the constructed PBPK model. Other research performed in rats [26] and humans [27] provided scant PK data and were therefore excluded from this study. Data were digitized from figures in the published literature.

Modeling input physiological parameters required in developing the PBPK model in mice (Table 1), rats (Supplementary Table 1), and humans (Supplementary Table 2) with cardiac output, tissue volume, and blood flow rate were obtained from published papers [28–30].

**Table 1:** Physiological and kinetic parameters for modeling iron PK and biodistribution in mice

Organs	Mouse (25 g)		
	Organ weight (g) ^a^	Organ volume (mL) ^b^	Blood flow rate (L/h) ^c^
Brain	0.47 ± 0.01	0.42	0.034
Bone	1.80 ± 0.25	3.50	0.042
Fat	0.31 ± 0.07	2.16	0.073
Gut	1.22 ± 0.19	1.06	0.146
Heart	0.14 ± 0.02	0.125	0.068
Kidney	0.38 ± 0.05	0.42	0.094
Liver	1.22 ± 0.10	1.375	0.167
Lung	0.13 ± 0.05	0.18	1.04
Muscle	13.42 ± 1.21	9.58	0.165
Skin	3.97 ± 0.73	4.125	0.06
Spleen	0.07 ± 0.01	0.092	0.012
Plasma	1.36 ± 0.03	1.09	-
Remainder	-	8.473	0.176

a-The organ weight of each tissue was obtained from literature data [28–30]. Data was expressed as mean ± SD

b-The organ volume in each tissue was obtained from literature data [28–30] and actual body and organ weight of mice used in the current study

c-The blood flow rate in each tissue was obtained from literature data [28–30] and actual body and organ weight of mice used in the current study

## PBPK model development

**Fig. 2:**
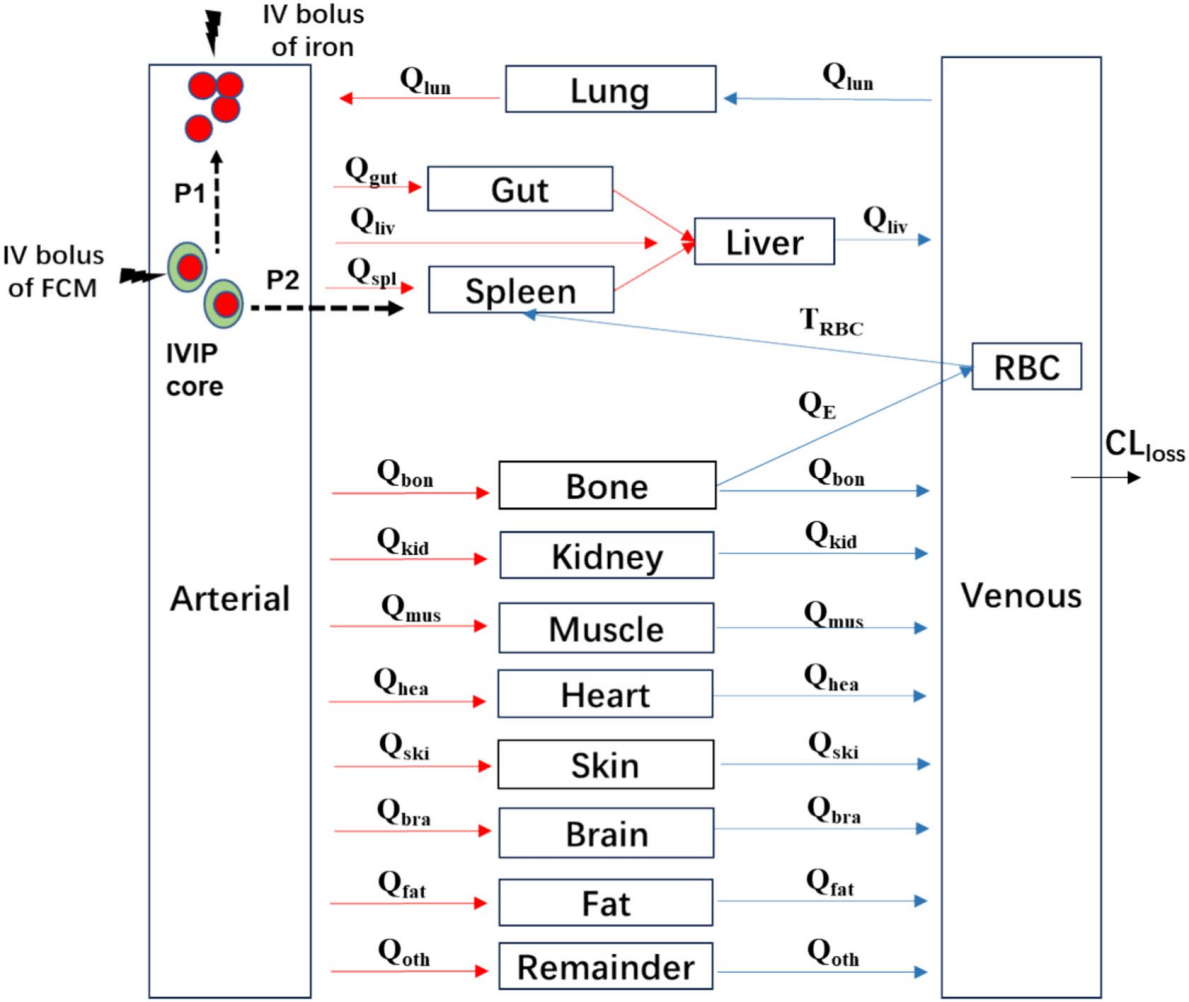
Schematic representation of the PBPK model for IV iron. Q = organ blood flow rate, Q_E_ = production rate of red blood cell (RBC) in the bone compartment by utilizing iron, T_RBC_ = lifespan of RBC, CL_loss_ = iron unavoidable daily loss rate, FCM = ferric carboxymaltose, IVIP = iron-carbohydrate complex preparation, FCM releases free iron through two pathways, including P1 direct (circulating IVIP-to-plasma) and P2 indirect (IVIP-to-macrophage-to-plasma) iron release

The whole-body PBPK model in mice was constructed with iron distribution data from thirteen tissues, including the red blood cells, heart, liver, spleen, lung, kidney, brain, bone, muscle, fat, skin, gut, and plasma (Fig. 2). Iron amounts in tissues such as the thymus were not measured and were thus set as the “remainder” compartment. The weight of the mice in this study was 25 g. Cardiac output (Q) was derived from the literature for a 20 g mouse: $\text{Q}2=\frac{BW2*Q1}{\text{BW}1}$, where BW1 is 20 g [31], and BW2 is 25 g [23]. The Q for the mouse applied in the model was 0.72 L/h.

The model's mathematical framework includes a mass balance equation for each compartment [32]. The following differential equations were utilized to characterize iron kinetics within the compartments:

(i) Other organs/tissues (including gut, heart, kidney, muscle, skin, lung, brain, fat, and the remainder):

1 $$V_O\cdot\frac{dC_O}{dt}=Q_O\cdot C_P-\frac{Q_O}{{KP}_O}\cdot C_O$$

where the abbreviations denote iron concentrations in organs (${C}_{O}$) and plasma (${C}_{P}$), organ blood flow rate (${Q}_{O}$), organ volume (${V}_{O}$), and organ to plasma partition coefficient (${KP}_{O}$). ${KP}_{O}$ measures the propensity of iron to exit an organ compartment into the plasma, indicating the degree of iron accumulation in a tissue relative to another under steady-state conditions [33].

(ii) Iron is mainly utilized to synthesize Hgb, a main protein in RBCs, in the bone marrow^10^, thus for the main effect compartment bone:

2 $$V_B\cdot\frac{dC_B}{dt}=Q_O\cdot C_P-(\frac{Q_B}{{KP}_B}+Q_E)\cdot C_B$$

3 $$\frac{dRBC}{dt}=Q_E\cdot C_B-\frac{RBC}{T_{RBC}}$$

where ${C}_{B}$ denotes iron concentration in the bone compartment, ${V}_{B}$ denotes the volumes of the bone compartment, ${Q}_{B}$ and ${KP}_{B}$ represent the blood flow rates of the bone and the tissue to plasma partition coefficient, respectively. ${Q}_{E}$ denotes the production rate of RBCs in the bone compartment by utilizing iron, while ${T}_{RBC}$ is the lifespan of RBC.

(iii) Because the RBCs recycling under steady state mainly occurs via macrophage-mediated erythrophagocytosis in the spleen, for the main iron recycling compartment spleen [9, 34]:

4 $$V_S\cdot\frac{dC_S}{dt}=Q_O\cdot C_p+\frac{RBC}{T_{RBC}}-\frac{Q_S}{{KP}_S}\cdot C_S$$

where ${C}_{S}$, ${V}_{S}$, ${Q}_{S}$, and ${KP}_{S}$ denote iron concentration in the spleen compartment, the volumes of the spleen compartment, the blood flow rates of the spleen, and the spleen to plasma partition coefficient, respectively.

(iv) Liver is the main storage compartment of iron [35]:

5 $$V_L\cdot\frac{dC_L}{dt}=Q_O\cdot C_p+\frac{Q_G}{{KP}_G}\cdot C_G+\frac{Q_S}{{KP}_S}\cdot C_S-\frac{Q_L}{{KP}_L}\cdot C_L$$

where ${C}_{L}$ and ${C}_{G}$ are the iron concentration in the liver and gut compartment, respectively, ${V}_{L}$ and ${V}_{G}$ represent the volumes of the liver and the gut compartment, respectively, ${Q}_{L}$ and ${Q}_{G}$ denote the blood flow rates of the liver and the gut, respectively, ${KP}_{L}$ and ${KP}_{G}$ denote the liver to plasma and the gut to liver partition coefficient, respectively.

(v) Plasma compartment:

6 $$V_p\cdot\frac{dC_p}{dt}=\sum(\frac{Q_O}{{KP}_O}\cdot C_O)-Q_{CO}\cdot C_P-{CL}_{Loss}\cdot C_P$$

where ${Q}_{CO}$ denotes the cardiac output, ${CL}_{Loss}$ represents iron's unavoidable daily loss rate^13^. Notably, net iron loss was not measured in the previous study^23^. The loss rate yields indirect information on iron fluxes through the physiologic exfoliation of cells after receiving IV iron administration.

In the data fitting, all physiological parameters, including blood flow rate and tissue volume, were collected from the literature and displayed in Table 1, while ${CL}_{Loss}$,${Q}_{E}$, $KP$ for all tissues, and ${T}_{RBC}$ were estimated in the model.

**Fig. 3:**
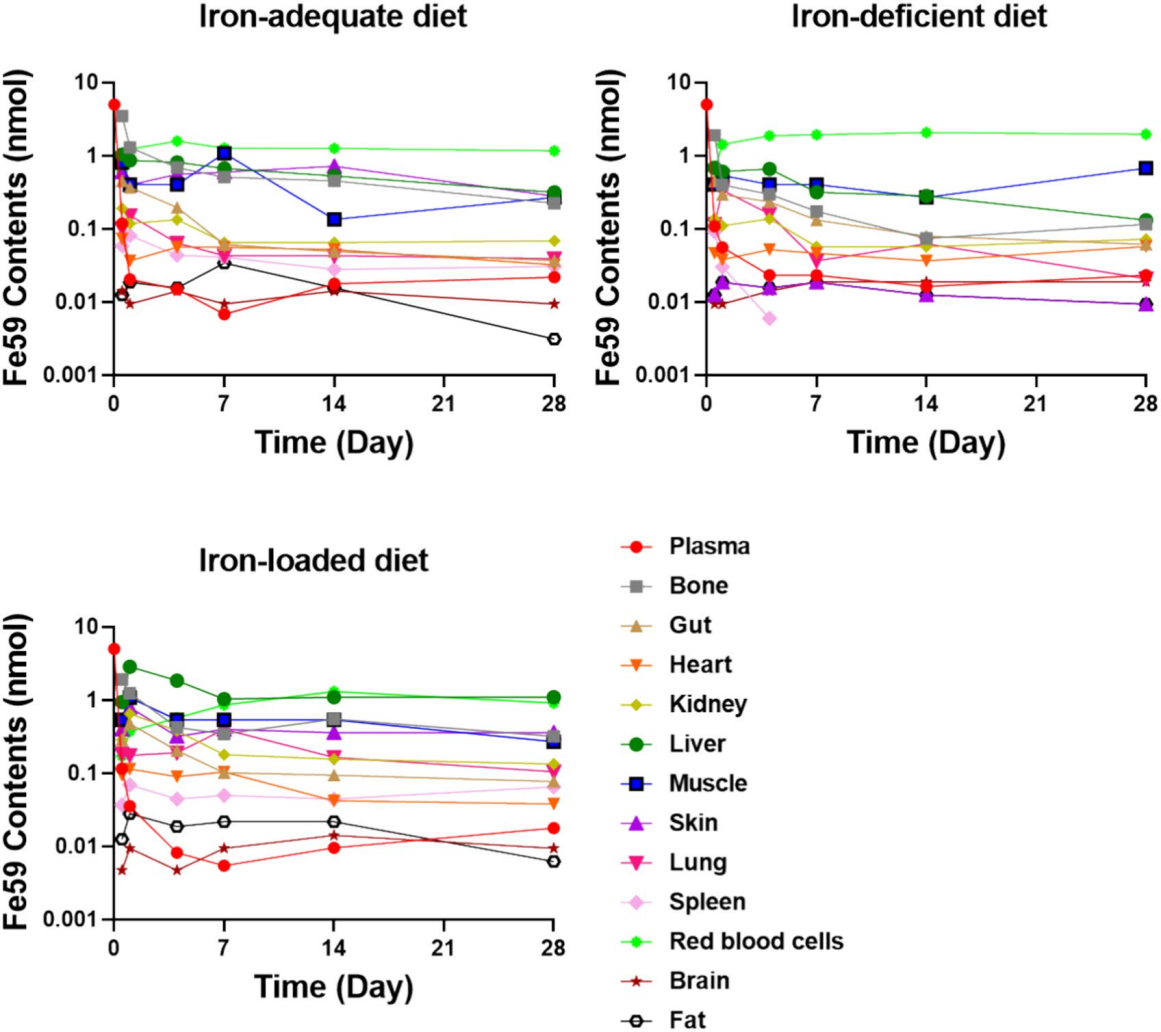
Fe^59^ contents in plasma and in 12 examined tissues (brain, red blood cells, fat, gut, spleen, heart, kidney, liver, lung, muscle, skin, bone) following intravenous bolus administration of 0.02 μmol/kg Fe^59^ in mice. Mean data was used at each time point

The concentration profiles of iron in plasma and 12 tissues are displayed in Fig. 3. All the data were fitted simultaneously in NONMEM 7.5. Different residual error models were used for different compartments, including an additive (7) or a proportional error model (8):

7 $$Y_{ij}={\overset\frown Y}_{ij}\cdot\left(1+\varepsilon_1\right)$$

8 $$Y_{ij}={\overset\frown Y}_{ij}+\varepsilon_2$$

Y_ij_ represents the observation of individual i at time t_j_, while Ŷ_ij_ denotes the individual prediction. The errors ε_1_ (proportional error) and ε_2_ (additive error) are normally distributed random variables with zero-mean and variance σ_1_^2^ and σ_2_^2^, respectively. Given that only mean data is available, a naïve pooled data method was adopted, treating observations from all individuals as if they originated from a single unique individual.

## Extrapolation from mice to rats and humans

The iron PBPK model established for mice was scaled to rats first and then to humans, considering interspecies variations in physiological parameters, such as organ volume and blood flow, because there is more data in the rat study^24^ compared with the human study^25^. Since FCM is an iron-carbohydrate complex preparation (IVIP) that is different from pure iron solution, the model was modified slightly to mimic the direct (IVIP-to-plasma) and indirect (IVIP-to-macrophage-to-plasma) iron release [36]. Initially, the direct release rate KR and the indirect release mediated by macrophage absorption rate KA were used to represent the two iron release pathways. However, the collected data is insufficient to differentiate these two parameters. In addition, the direct iron release is relatively minimal (approximately 0.1% of the total iron dose)^16^. To simplify the model, the direct release was removed to reduce the KR parameter. Since macrophage-mediated FCM absorption mainly occurs at the spleen, to further simplify the model, the FCM absorption rate KA was incorporated as the spleen blood flow rate. These two pathways were expressed as dashed lines (Fig. 2).

The other model structure and equations remain consistent. In the PBPK model for rats, the $KP$ for the same type of tissues that were not detected in the experiment, ${CL}_{Loss}$ values were assumed to be identical across tissues in mice, because the data is insufficient to estimate these parameters. The $KP$ values for the tissues that were detected in the experiment, including bone, liver, heart, muscle, kidney, and spleen, were estimated using the data in these tissues obtained from the literature^24^. The other physiological parameters in rats were fixed to the literature values (Supplementary Table 1).

In humans, mean serum iron data from 4 dose levels were digitized and used for extrapolation, including 100, 500, 800, and 1000 mg. The KP for the same type of tissues and CL_Loss_ values were assumed to be identical among tissues in rats. Human physiological parameters were fixed to the literature values (Supplementary Table 2). ${Q}_{E}$ for humans was scaled from mice to humans using an allometric Eq. ^14^. Different allometric equations were investigated, including body weight-based scaling and RBC lifespan-based scaling. Eventually, the RBC lifespan-based scaling was used and described as:

9 $${Q}_{E(human)}={Q}_{E(mouse)}\cdot {(\frac{{{T}_{RBC}}_{human}}{{{T}_{RBC}}_{mouse}})}^{b}$$

T_RBC_ used in the human simulation was 120 days. A fixed value of 0.75 was employed for the power function b for the allometric relationship^14^. Body weights of 0.345 and 73 kg were assigned to rats and humans respectively. The cardiac output of rats and humans were 6.624 and 336 L/h respectively [28–30].

## Data analysis

The ADVAN15 subroutine was used for solving the ordinary differential equations, while parameter estimation employed the first-order conditional estimation method with interaction (FOCEI).

## Results

### Data collection of the PBPK model

The input parameters of iron for the PBPK model in mice are given in Table 1. As shown in Fig. 3, iron exhibited widespread distribution in most tissues after IV injection, with exceptions in fat, brain, and skin. Twelve hours after injection, ^59^Fe was very rapidly cleared from the plasma concentration. Bone is the major consumer organ of iron for the synthesis of RBCs/Hgb. Under different iron statuses, the iron content showed different kinetic behavior. In iron-deficient mice, ^59^Fe-content in the bone notably declined post-administration, consistently lower than in iron-adequate mice. These findings align with the rapid increase of ^59^Fe in RBCs (Fig. 3), suggesting that iron was consumed for the production of Hgb in the RBCs. As expected, the liver functioned as the principal organ for iron storage [35], with initial accumulation followed by redistribution over time, demonstrating significant differences across iron statuses. Moreover, the impact of iron deficiency on other organs was particularly marked, as a substantial proportion of ^59^Fe shifted towards circulating RBCs over time (Fig. 3).

The data of iron for the PBPK model in rats include the concentration of iron in serum, bone, heart, kidney, liver, spleen, and skeletal muscle after receiving a single IV dose of 30 mg Fe/kg of FCM^24^. The human data contains serum iron concentration after receiving four single ascending doses (100, 500, 800, and 1000 mg) of IV iron as FCM^25^.

## The model fits the literature data in mice well

The PBPK model is displayed in Fig. 2. The model employed a perfusion-limited description in which the behavior of iron is similar to distribute freely and instantaneously across the cell membrane without a diffusion barrier, because IV iron administration results in transferrin saturation and dangerously high concentrations of non-transferrin-bound iron (NTBI) in plasma. Thus, other pathways, such as Zrt/Irt-like protein 14 and ferritin, can take up NTBI very efficiently, for salvaging and reutilizing iron to avoid toxicity. In this situation, the behavior of iron enter tissues fits the perfusion-limited model^9^. In this model, the rate of distribution is primarily limited by the blood flow rate.

**Fig. 4:**
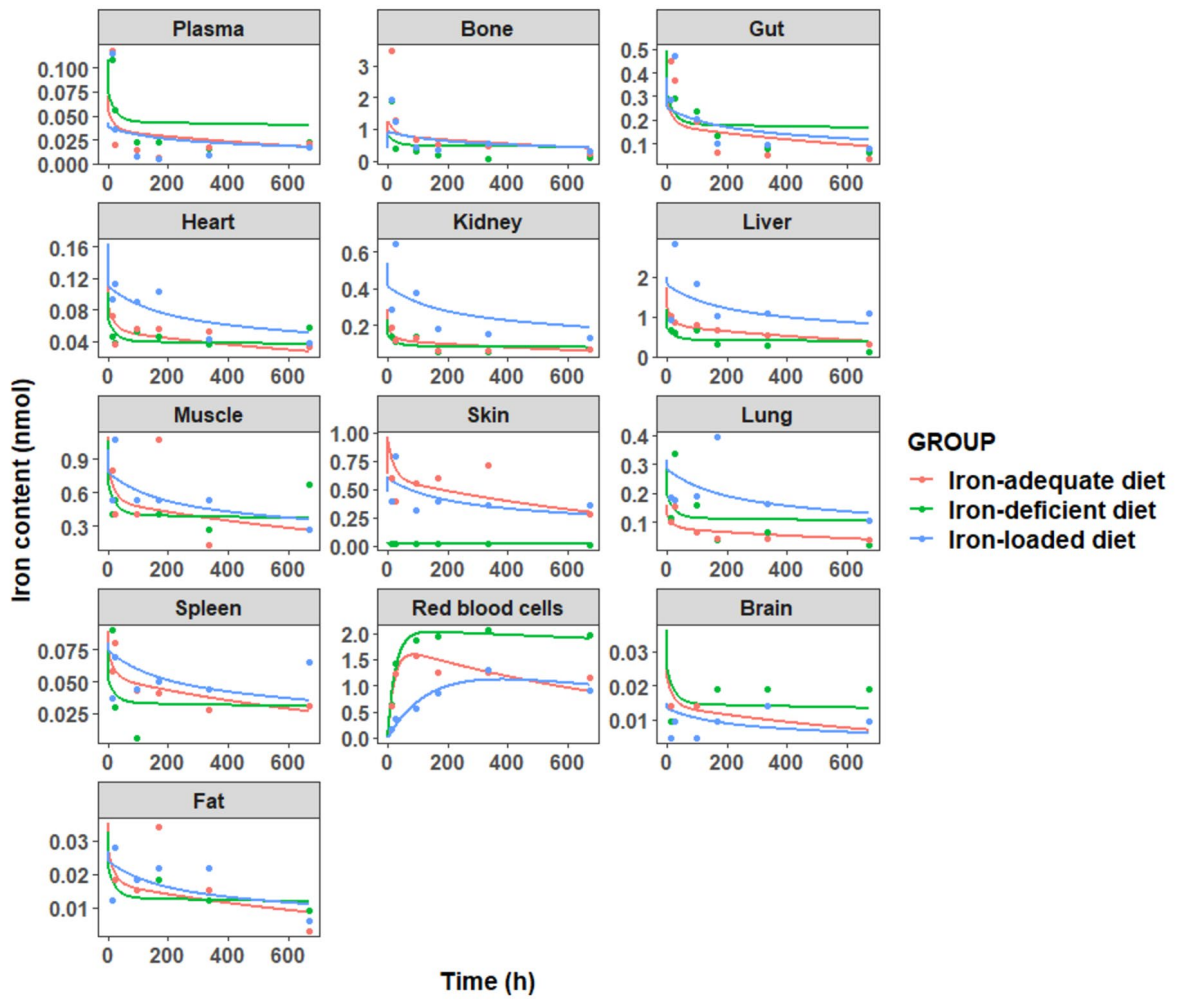
Observed (dots) and predicted (lines) iron concentrations in plasma and various organs/tissues in mice. Each dot represents the mean of the observations digitized from the literature [18, 23]. Solid lines are the fit predictions based on the proposed PBPK model

As shown in Fig. 4, the proposed PBPK model effectively characterized the iron concentration–time profiles in plasma and tissues, with model predictions closely aligning with the experimentally observed data. The goodness-of-fit diagnostic plots (Supplementary Figure S1) demonstrated a random normal scatter around the identity line, effectively capturing the overall trend and observed variability. This confirmed the model's adequacy in accurately describing the data. The estimated iron PK parameters $KP$, ${CL}_{Loss}$, ${Q}_{E}$, and ${T}_{RBC}$ in mice are shown in Table 2. Overall, the estimated lifespan ${T}_{RBC}$ was shorter in the iron deficiency mice. This is reasonable because the increased Hgb autoxidation and subsequent production of ROS can explain the reduced RBC lifespan associated with IDA [37]. High ${Q}_{E}$ values were estimated in IDA mice (0.434 × 10^–3^ L/h) compared with the normal mice (0.217 × 10^–3^ L/h) and iron-loaded mice (0.0388 × 10^–3^ L/h), while low $KP$ values were estimated for bone, liver, spleen, heart, and kidney compartments in IDA mice compared with the normal and iron-loaded mice, indicating that iron was consumed for the production of Hgb in the RBCs during IDA situation, consistently with the high tissue concentrations of iron measured in iron-loaded mice. On the contrary, high loss rate of iron from plasma after IV iron administration CL_loss_ value was estimated in iron-loaded mice (1.184 × 10^–4^ L/h) and normal mice (1.647 × 10^–4^ L/h) compared with the IDA mice (0.124 × 10^–4^ L/h), consistently with the high Q_E_ values estimated in IDA mice and further demonstrating that iron was consumed for the production of Hgb in the RBCs during IDA situation.

**Table 2:** Iron pharmacokinetic parameter estimation and precision (RSE) in mouse plasma and tissues using the proposed PBPK model

Organs	Parameters (Unit)	PBPK estimated (RSE%)		
		Iron-deficient diet	Iron-adequate diet	Iron-loaded diet
Plasma	CL_loss_ (× 10^–4^ L/h)	0.124 (6.19)	1.647 (0.016)	1.184 (0.035)
Brain	KP_bra_	1.075 (0.045)	1.087 (0.011)	0.9304 (0.048)
Bone	KP_bon_	3.635 (0.35)	7.703 (0.035)	7.753 (0.038)
Fat	KP_fat_	0.186 (0.042)	0.258 (0.011)	0.328 (0.025)
Gut	KP_gut_	5.213 (0.14)	5.349 (0.037)	6.979 (0.033)
Heart	KP_hea_	10.08 (0.092)	13.83 (0.096)	25.88 (0.043)
Kidney	KP_kid_	6.541 (0.045)	9.223 (0.013)	29.06 (0.042)
Liver	KP_liv_	9.38 (0.10)	18.11 (0.081)	38.53 (0.036)
Lung	KP_lun_	17.86 (0.023)	13.7 (0.001)	45.24 (0.027)
Muscle	KP_mus_	1.288 (0.07)	1.764 (0.014)	2.394 (0.034)
Red blood cells	Q_E_ (× 10^–3^ L/h)	0.434 (0.29)	0.217 (0.012)	0.0388 (0.089)
	T_RBC_ (h)	41.53 (0.067)	34.44 (0.021)	197.6 (0.016)
Skin	KP_ski_	0.101 (0.042)	4.651 (0.010)	4.252 (0.027)
Spleen	KP_spl_	10.13 (0.06)	16.34 (0.068)	23.93 (0.046)
Remainders	KP_rem_	4.93 (0.079)	2.30E-09 (2.78)	3.06E-08 (8.16)

Taken together, the results have indicated that the present PBPK model could predict PK profiles with reasonable accuracy and estimate the biodistribution of iron in mice under different iron statuses.

## Extrapolation of the PBPK model from mice to rats

Before extrapolating this PBPK model to humans, we assessed its capability to predict PK and biodistribution of iron after receiving FCM treatment in rats.

**Fig. 5:**
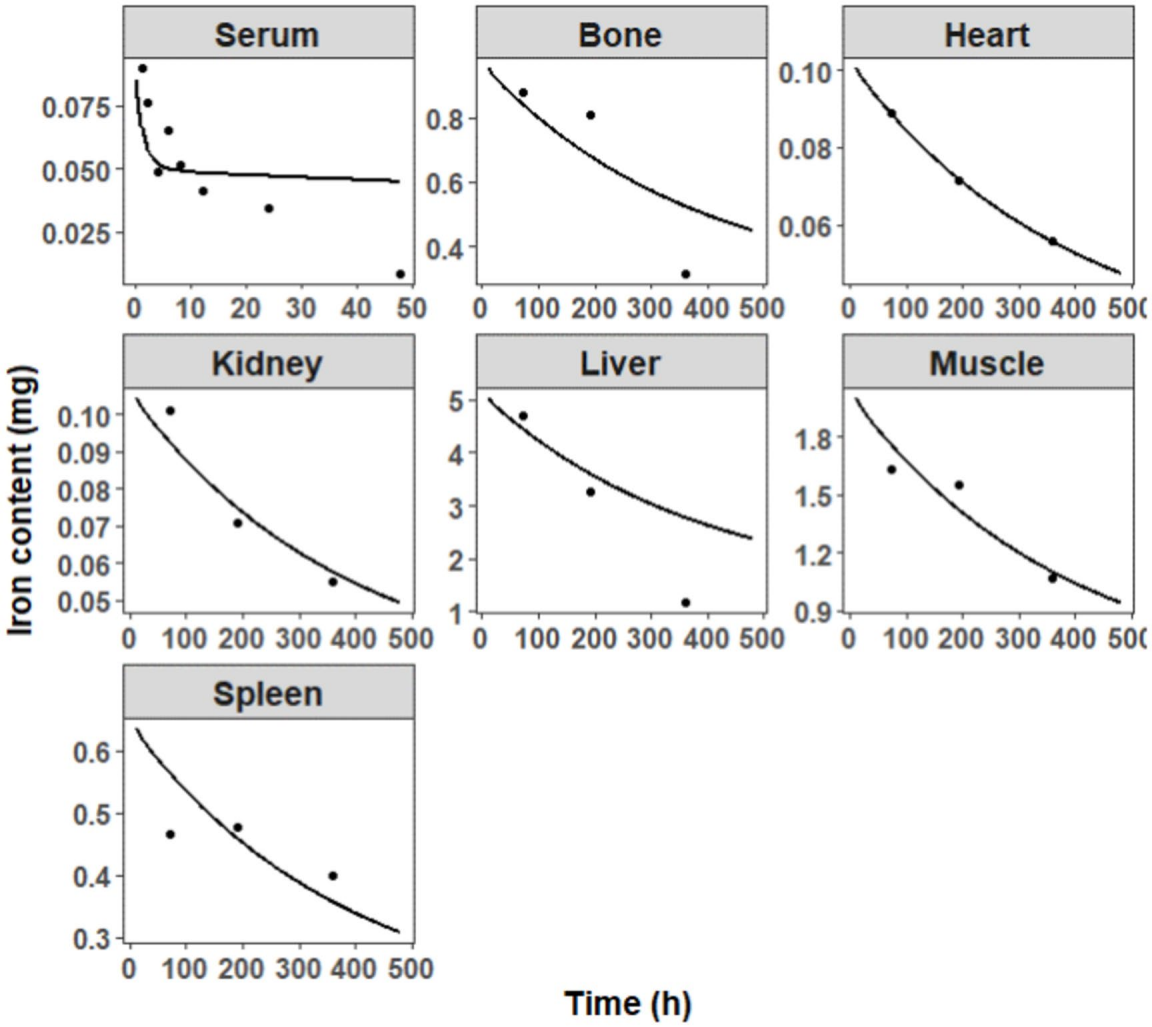
Observed (dots) and predicted (lines) iron concentrations in serum and various organs/tissues in rats. Each dot represents the mean of the observations digitized from the literature [24]. Solid lines are the fit predictions based on the proposed PBPK model

Time-concentration profiles of iron in several rat tissues were obtained from a previous study^24^, in which rats with IDA received 30 mg Fe/kg of FCM. As shown in Fig. 5, although the iron concentration in the serum was slightly overestimated to a lesser degree after 20 h, the model simulations adequately approximated the observed data in bone, heart, kidney, liver, muscle, and spleen. The estimated $KP$ values for bone, liver, spleen, heart, muscle, and kidney compartments in IDA rats were 8.64, 59.4, 147.9, 17.64, 2.28, and 6.41, respectively.

## Extrapolation of PBPK model to humans

Among the literature documenting PK data of FCM in humans, only serum iron PK profiles were reported^25^. Patients with IDA received single ascending IV doses of FCM, including 100, 500, 800, and 1000 mg Fe. A human PBPK model was constructed using parameters derived from human physiology typical for a 70 kg healthy adult. The iron serum PK profile was collected to evaluate the predictive capability of the model.

**Fig. 6:**
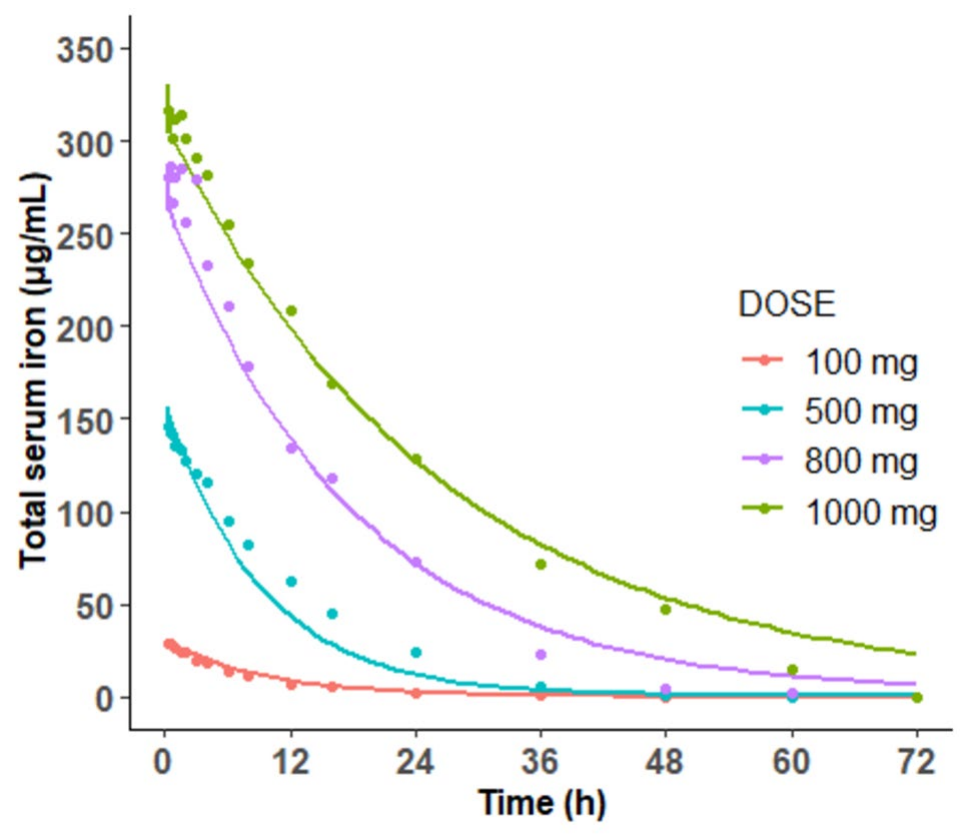
Observed (dots) and model-simulated (lines) iron concentrations in serum in humans after an IV dose of 100, 500, 800, or 1000 mg. Each dot represents the mean of the observations digitized from the literature [25]. Solid lines are the simulations based on the proposed PBPK model

The observed human data were overlaid with the simulation results following identical FCM dosing regimens. The model effectively predicted the human serum PK profile of iron, with simulated serum iron concentrations generally aligning well with observed values in patients with IDA following IV dose regimens of FCM (Fig. 6).

## Discussion

Anemia represents a significant global public health concern. Iron deficiency stands as the predominant cause of anemia, particularly in CKD patients. Expert consensus regarding IV iron therapy for IDA indicates robust evidence supporting its efficacy and safety across various acute and chronic conditions. The observed clinical endpoint provides reassurance regarding the safety and potential benefits of high-dose IV iron therapy, targeting higher ferritin and TSAT concentrations than those typically recommended by current guidelines [38]. Nonetheless, guidelines for iron management in CKD patients remain somewhat outdated, contradictory, and grounded in limited evidence. Furthermore, findings from observational studies have yielded conflicting results regarding the long-term safety of IV iron therapy, particularly concerning risks such as mortality and infection [39]. Studies indicated that a significant proportion of hemodialysis patients receiving IV iron administration exhibit hepatic iron overload on magnetic resonance imaging [40], while iron accumulation in the heart and liver would generate ROS and damage the tissues [3, 41, 42]. In addition, while serum ferritin concentration and transferrin saturation are useful for guiding clinical decisions regarding IV iron therapy, determining optimal iron status precisely in individual patients with CKD remains clinically challenging and necessitates further investigation [43]. It is an urgent need to quantify the exposure–response relationship of IV iron.

Understanding the PK profiles of iron in various organs is crucial for evaluating its safety. The mechanistic PBPK model of iron incorporates regulation mechanisms and tissue transport dynamics across the organ systems of the entire body. Utilizing this PBPK model enables the evaluation and prediction of iron distribution dynamics in diverse tissues, thereby enhancing comprehension of the relationship between tissue exposure and both drug safety and efficacy [14].

In the present study, we first constructed a PBPK model capable of capturing the concentration–time profiles of iron in plasma and various organs following an IV administration of 0.2 μmol ^59^Fe/kg in mice under different iron statuses. The results showed that the supplemented iron mainly consumed for the synthesis of Hgb that eventually flushed into the RBCs if the mice were iron deficient. In contrast, the supplemented iron was mainly stored in the liver and was later redistributed if the mice were iron-adequate or iron-rich. These findings align with the mechanism of action of iron, as the iron will be used for the production of Hgb and liver is the main iron storage compartment [35].

The established PBPK model was successfully scaled from mice to rats and humans, considering interspecies variations in physiology, to predict human serum iron concentrations. FCM is an IVIP and has two pathways to release iron from the IVIP core, which is different from pure iron solution [36]. However, the collected data is insufficient to differentiate these two pathways. To simplify the model, the direct release was removed because the direct iron release is relatively minimal (approximately 0.1% of the total iron dose)^16^, and the macrophage-mediated FCM absorption rate was incorporated as the spleen blood flow rate. The data in rats were used for model development and validation first because the data in rats is richer than humans. Although the model fitting in serum was slightly overestimated due to limited data at later times, the model fitting in other organs was good. Eventually, the whole-body PBPK model successfully extrapolated the PK of FCM from mice and rats to humans. Iron serum concentrations in humans decrease rapidly. Given the widespread distribution of iron throughout tissues, serum concentration may not reliably reflect iron exposure at specific target sites or organs where toxicity may occur. Our comprehensive semi-mechanistic whole-body PBPK model not only predicts iron concentrations in serum but also in multiple organs. This capability suggests potential clinical utility in evaluating the efficacy and safety of FCM, offering insights for optimizing therapeutic strategies.

This study has limitations that merit further discussion and exploration in future research. First, iron tissue samples were only obtained up to a single dose. Therefore, caution is advised in interpreting the model results for extrapolation to multiple doses over extended time periods. Moreover, the iron tissue samples were only collected up to mean data as the individual data were not available from the literature, which may have some bias during the simultaneous fitting of all thirteen tissues. In addition, the time point in the terminal phase is sparse, which may also lead to some bias. Further investigation is warranted to evaluate individual level data and iron tissue distribution after multiple doses over longer periods. Second, the molecular mechanism was simplified in this model. RES system should be more complex as iron uptake was mediated by transferrin, which was not considered in the current PBPK model to avoid overparameterization due to limited data. Third, the daily loss of iron can be achieved through skin and enteric desquamation and minor blood losses, the current study only included CL_Loss_ to represent iron unavoidable loss rate through the physiologic exfoliation of cells after receiving IV iron administration due to limited data. In addition, different iron preparations should have different PK profiles due to different release rates and absorption rates, which warrant further investigations.

## Conclusion

In conclusion, we presented a novel whole-body PBPK model of iron which is capable of describing the PK and biodistribution of pure iron and FCM as evidenced by reproducing the observed PK data. The model also provides mechanistic insights regarding the mechanism of action of iron by integrating the production of RBCs and the macrophage-mediated recycling of senescent RBCs. Our model may serve as a valuable tool for optimizing the dosing regimen of the FCM therapy through simulations of iron serum concentration and tissue accumulation, and have potential clinical applications for evaluating the efficacy and safety of iron preparations.

## Data availability statement

Raw data and the model code are available from the corresponding author on request.

## Supplementary Information

Below is the link to the electronic supplementary material.

Supplementary file1 (DOCX 404 KB)

## Data Availability

All data generated or analysed during this study are included in this published article [and its supplementary information files].
