# Supplementary material for: A whole-body mechanistic physiologically-based pharmacokinetic modeling of intravenous iron
Source: Drug Deliv Transl Res. 2024 Jul 24;15(4):1109–20. doi: 10.1007/s13346-024-01675-x (PMC11870943; doi:10.1007/s13346-024-01675-x)
Supplement: Supplementary file 1 — Supplementary file1 (DOCX 404 KB) [file 13346_2024_1675_MOESM1_ESM.docx]

**
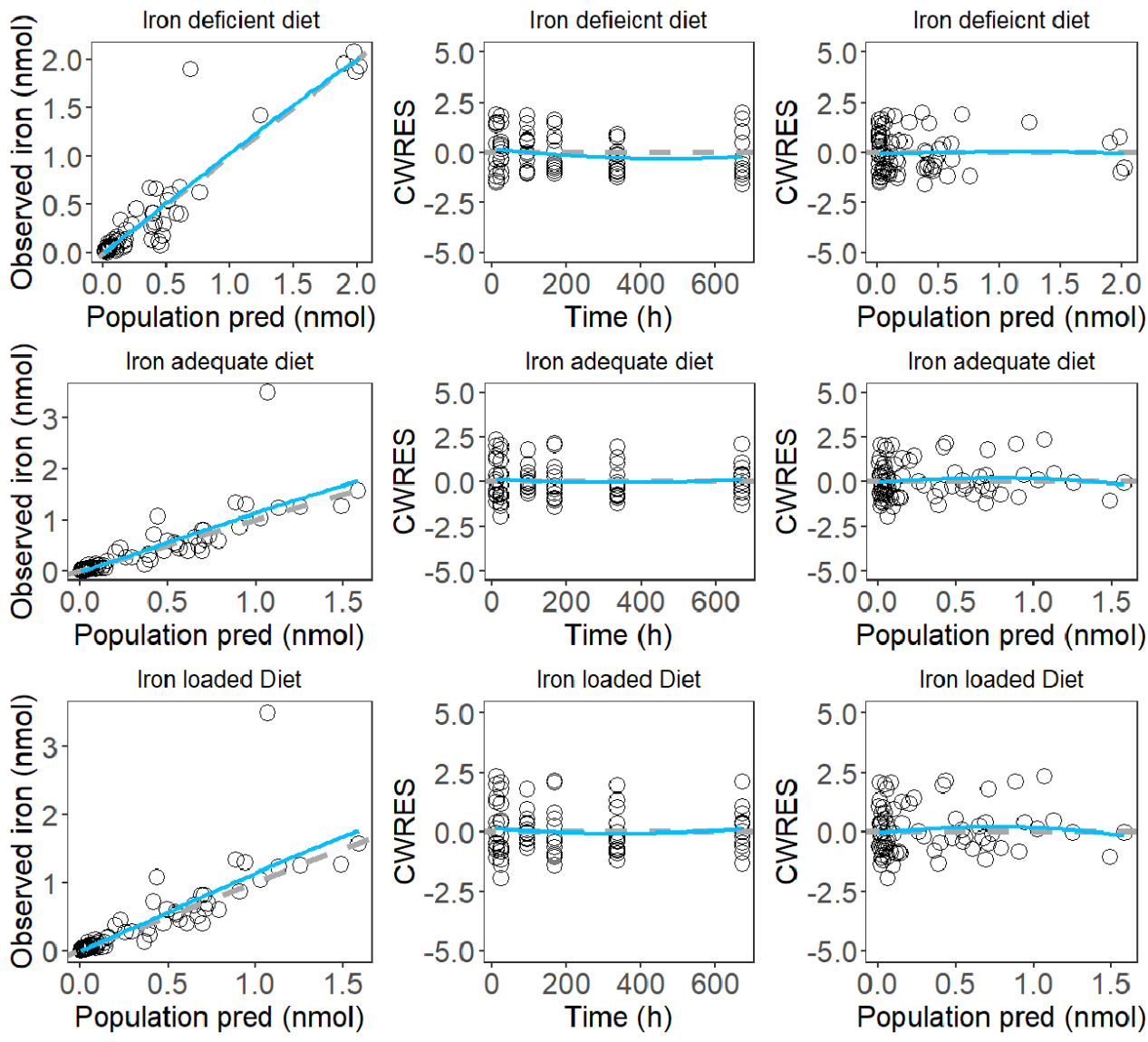
Fig. S1**. General goodness-of-fit of the final model for iron deficient diet (top panels), iron adequate diet (middle panels), and iron loaded diet (bottom panels) in mice. Including the observed data vs. population predictions (left), the conditional weighted residual (CWRES) vs. time (middle), and CWRES vs. population predictions (right). The blue lines are the LOESS smooth lines. The gray diagonal (top panels) and horizontal (bottom panels) lines are the identity and zero lines, respectively.

**Supplementary Table 1**. Physiological and kinetic parameters for modeling iron PK and biodistribution in rats.

| **Organs** | **Rat (345 g)** | | |
| --- | --- | --- | --- |
|  | **Organ weight (g) ^a^** | **Organ volume (mL) ^b^** | **Blood flow rate (L/h) ^c^** |
| **Brain** | - | 11.17 | 0.132 |
| **Bone** | 20.7 | 17.13 | 0.168 |
| **Fat** | - | 10.84 | 0.0265 |
| **Gut** | - | 10.84 | 0.498 |
| **Heart** | 1.38 | 0.87 | 0.260 |
| **Kidney** | 2.76 | 2.49 | 0.611 |
| **Liver** | 13.8 | 11.17 | 0.782 |
| **Lung** | - | 1.08 | 6.624 |
| **Muscle** | 138 | 132.28 | 0.134 |
| **Skin** | - | 43.37 | 0.386 |
| **Spleen** | 1.035 | 0.65 | 0.0417 |
| **Serum** | - | 7.8 | - |
| **Remainder** | - | 27.27 | 3.584 |

Notes:

a-The organ weight of each tissue was obtained from literature data^28-30^. Data was expressed as mean value.

b-The organ volume in each tissue was obtained from literature data^28-30^ and actual body and organ weight of rats used in the current study

c-The blood flow rate in each tissue was obtained from literature data^28-30^ and actual body and organ weight of rats used in the current study

**Supplementary Table 2**. Physiological and kinetic parameters for modeling iron PK and biodistribution in humans.

| **Organs** | **Human (73 kg)** | |
| --- | --- | --- |
|  | **Organ volume (L) ^a^** | **Blood flow rate (L/h) ^b^** |
| **Brain** | 1.4 | 40.32 |
| **Bone** | 10.5 | 16.8 |
| **Fat** | 12.5 | 16.8 |
| **Gut** | 1.2 | 50.4 |
| **Heart** | 0.33 | 13.44 |
| **Kidney** | 0.31 | 63.84 |
| **Liver** | 1.8 | 21.84 |
| **Lung** | 0.47 | 336 |
| **Muscle** | 30 | 56.99 |
| **Skin** | 3.3 | 16.8 |
| **Spleen** | 0.18 | 10.08 |
| **Serum** | 3 | - |
| **Remainder** | 8.01 | 28.69 |

Notes:

a-The organ volume in each tissue was obtained from literature data^28-30^ and actual body and organ weight of humans used in the current study.

b-The blood flow rate in each tissue was obtained from literature data^28-30^ and actual body and organ weight of humans used in the current study.

NONMEM code

$SIZES LVR=160

$PROBLEM IRON PBPK

$INPUT ID TIME DV DOSE GROUP DROP CMT AMT MDV

$DATA IRONPBPK03.csv IGNORE=@

$SUBROUTINES ADVAN15 TOL=12

$MODEL

NCOMP=14

COMP = (ARTERIAL); COMP(1)-Plasma

COMP = (BON); COMP(2)-Bone

COMP = (GUT); COMP(3)-Gut (stomach, small & large intestine)

COMP = (HRT); COMP(4)-Heart

COMP = (KID); COMP(5)-Kidney

COMP = (LIV); COMP(6)-Liver

COMP = (MUS); COMP(7)-Muscle

COMP = (SKN); COMP(8)-Skin

COMP = (LUN); COMP(9)-Lung

COMP = (SPL); COMP(10)-Spleen

COMP = (RBC); COMP(11)-red blood cells

COMP = (BRA); COMP(12)-Brain

COMP = (ADI); COMP(13)-Fat

COMP = (ROB); COMP(14)-Rest Of Body

$PK

CLG = (THETA(1)/10000)*EXP(ETA(1)) ; Daily loss rate (L/h)

KP1 = (THETA(2))*EXP(ETA(2)) ; Bone partition coefficient

KP2 = (THETA(3))*EXP(ETA(3)) ; Gut

KP3 = THETA(4)*EXP(ETA(4)) ; Heart

KP4 = THETA(5)*EXP(ETA(5)) ; Kidney

KP5 = (THETA(6))*EXP(ETA(6)) ; Liver

KP6 = (THETA(7))*EXP(ETA(7)) ; Muscle

KP7 = THETA(8) ; Skin

KP8 = THETA(9) ; Lung

KP9 = THETA(10) ; Spleen

KP10 = THETA(11) ; ROB

QE = THETA(12)/1000 ; production rate of RBC

TRBC = THETA(13) ; Recycle rate of RBC

KP11 = THETA(14) ; Brain

KP12 = THETA(15) ; Fat

; PHYSIOLOGICAL MODEL PARAMETERS for a 25 g standard weight mouse

; CALCULATING TISSUE BLOOD FLOOWS

; Relationship between cardiac output (CO) and body weight (BW)

BW = 0.025 ; body weight of mice in kilograms

CO = 0.275*(BW**(0.75)) ; L/min [Brown Arms and Travis (1988)] BW equals body weight in kilograms.

; -------------- Blood flow rates ----------------

QCO=60*CO ; cardiac output (L/h)

QADI=0.07*QCO ; Adipose blood flow (L/h)

QBON=0.0407*QCO ; Bone blood flow

QBRA=0.033*QCO ; Brain blood flow

QGUT=0.1408*QCO ; Gut blood flow (L/h)

QHEA=0.066*QCO ; Heart blood flow

QKID=0.091*QCO ; Kidney blood flow

QLIV=0.161*QCO ; Total liver blood flow

Q_HA = 0.02*CO ; HA = hepatic artery

Q_PV = 0.141*CO ; PV = portal vein

QMUS=0.159*QCO ; Muscle blood flow

QSKI=0.058*QCO ; Skin blood flow

QLUN= QCO ; Lung blood flow

QSPL=0.0112*QCO ; Spleen blood flow

QROB=QCO – QBON –QHEA – QKID – QLIV – QMUS – QSKI– QADI - QBRA ; Rest of Body blood flow

; -------------- Tissue volumes (real volumes) ----------------

VBODY=0.0326 ; body volume (L) of mice

VADI=2.59*25/(30*1000) ; Adipose volume (L)

VBON=0.1073*VBODY ; Bone volume (L)

VBRA=0.50*25/(30*1000) ; Brain volume (L)

VGUT=1.27*25/(30*1000) ; Gut volume (L)

VHEA=0.15*25/(30*1000) ; Heart volume (L)

VKID=0.5*25/(30*1000) ; Kidney volume (L)

VLIV=1.65*25/(30*1000) ; Liver volume (L)

VMUS=11.5*25/(30*1000) ; Muscle volume (L)

VSKI=4.95*25/(30*1000) ; Skin volume (L)

VLUN=0.22*25/(30*1000) ; Lung volume (L)

VSPL=0.11*25/(30*1000) ; Spleen volume (L)

VBLD=1.2*25/(27.4*1000) ; Plasma volume (L)

VROB=VBODY-VBON-VGUT-VHEA-VKID-VLIV-VMUS-VSKI-VLUN-VSPL-VBLD-VADI-VBRA ; Rest of Body volume (L)

$DES

C1=A(1)/VBLD;

C2=A(2)/VBON;

C3=A(3)/VGUT;

C4=A(4)/VHEA;

C5=A(5)/VKID;

C6=A(6)/VLIV;

C7=A(7)/VMUS;

C8=A(8)/VSKI;

C9=A(9)/VLUN;

C10=A(10)/VSPL;

;C11=A(11)/VRBC;

C12=A(12)/VBRA;

C13=A(13)/VADI;

C14=A(14)/VROB;

DADT(1) = -(QROB + QBON + QHEA + QKID + QLIV + QMUS + QSKI + CLG + QBRA + QADI) *C1 + (QBON/KP1)*C2 + (QHEA/KP3)*C4 + (QKID/KP4)*C5 + (QLIV/KP5)*C6 + (QMUS/KP6)*C7 + (QSKI/KP7)*C8 + (QROB/KP10) * C14 + (QBRA/KP11) * C12 + (QADI/KP12) * C13 ; arterial comp

DADT(2) = (QBON)*C1 -(QBON/KP1 + QE) * C2 ; eq Bone comp.

DADT(3) = (QGUT)*C1-(QGUT/KP2)*C3 ; eq Gut comp.

DADT(4) = (QHEA)*C1-(QHEA/KP3)*C4 ; eq Heart comp.

DADT(5) = (QKID)*C1-(QKID/KP4)*C5 ; eq Kidney comp.

DADT(6)=(QLIV- QGUT- QSPL) * C1 + QGUT/KP2 * C3 + QSPL/KP9 * C10 -(QLIV/KP5)*C6 ; Liver comp.

DADT(7) = (QMUS)*C1-(QMUS/KP6)*C7 ; eq. Muscle comp.

DADT(8)= (QSKI)*C1-(QSKI/KP7)*C8 ; eq Skin comp.

DADT(9)= QCO*C1-(QCO/KP8)*C9 ; eq Lung comp.

DADT(10)= (QSPL)*C1+A(11)/TRBC-(QSPL/KP9)*C10 ; eq. Spleen comp.

DADT(11)= QE*C2 -A(11)/TRBC ; eq RBC.

DADT(12) = (QBRA)*C1-(QBRA/KP11)*C12 ; eq. Brain comp.

DADT(13)= (QADI)*C1-(QADI/KP12)*C13 ; eq fat comp.

DADT(14)= QROB*C1-(QROB/KP10)*C14 ; eq Rest Of Body comp.

$ERROR

IF (CMT.EQ.1) THEN

IPRED=A(1)

Y=IPRED+IPRED*EPS(1)+EPS(14)

ELSE

ENDIF

IF (CMT.EQ.2) THEN

IPRED=A(2)

Y=IPRED+IPRED*EPS(2)+EPS(15)

ELSE

ENDIF

IF (CMT.EQ.3) THEN

IPRED=A(3)

Y=IPRED+IPRED*EPS(3)+EPS(16)

ELSE

ENDIF

IF (CMT.EQ.4) THEN

IPRED=A(4)

Y=IPRED+IPRED*EPS(4)+EPS(17)

ELSE

ENDIF

IF (CMT.EQ.5) THEN

IPRED=A(5)

Y=IPRED+IPRED*EPS(5)+EPS(18)

ELSE

ENDIF

IF (CMT.EQ.6) THEN

IPRED=A(6)

Y=IPRED+IPRED*EPS(6)+EPS(19)

ELSE

ENDIF

IF (CMT.EQ.7) THEN

IPRED=A(7)

Y=IPRED+IPRED*EPS(7)+EPS(20)

ELSE

ENDIF

IF (CMT.EQ.8) THEN

IPRED=A(8)

Y=IPRED+IPRED*EPS(8)+EPS(21)

ELSE

ENDIF

IF (CMT.EQ.9) THEN

IPRED=A(9)

Y=IPRED+IPRED*EPS(9)+EPS(22)

ELSE

ENDIF

IF (CMT.EQ.10) THEN

IPRED=A(10)

Y=IPRED+IPRED*EPS(10)+EPS(23)

ELSE

ENDIF

IF (CMT.EQ.11) THEN

IPRED=A(11)

Y=IPRED+IPRED*EPS(11)+EPS(24)

ELSE

ENDIF

IF (CMT.EQ.12) THEN

IPRED=A(12)

Y=IPRED+IPRED*EPS(12)+EPS(25)

ELSE

ENDIF

IF (CMT.EQ.13) THEN

IPRED=A(13)

Y=IPRED+IPRED*EPS(13)+EPS(26)

ELSE

ENDIF

IRES=DV-IPRED

$THETA

(0,0.3) ; 1 CLG

(0,1) ; 2 KP1

(0,1) ; 3 KP2

(0,1) ; 4 KP3

(0,1) ; 5 KP4

(0,1) ; 6 KP5

(0,1) ; 7 KP6

(0,0.1) ; 8 KP7

(0,1) ; 9 KP8

(0,5) ; 10 KP9

(0,0.01) ; 11 KP10

(0,1) ; 12 QE

(0,60) ; 13 TRBC

(0,1) ; 14 KP11

(0,1) ; 15 KP12

$OMEGA

0 FIX

0 FIX

0 FIX

0 FIX

0 FIX

0 FIX

0 FIX

$SIGMA

0.1

0.5

0.1

0 FIX

0 FIX

0 FIX

0 FIX

0.1

0.1

0.1

0 FIX

0.1

0 FIX

0 FIX

0 FIX

0 FIX

0.1

0.1

0.1

0.1

0 FIX

0 FIX

0 FIX

0.1

0 FIX

0.1

$EST MAXEVAL=9999 METHOD=1 PRINT=1 SIGDIGITS=3 INTERACTION NOABORT NOSIGMABOUNDTEST NOOMEGABOUNDTEST NOTHETABOUNDTEST MSFO=RUN1.MSF

$COV PRINT=E

$TABLE ID TIME DV AMT MDV DOSE GROUP CMT IPRED IRES NOPRINT ONEHEADER FILE=mytab1 FORMAT=s1PE17.9

DATA FILES

Iron deficiency diet mice

| Time  (Days) | Fe59 amount in tissues (nmol) | | | | | | | | | | | | |
| --- | --- | --- | --- | --- | --- | --- | --- | --- | --- | --- | --- | --- | --- |
|  | Plasma | Bone | Gut | Heart | Kidney | Liver | Muscle | Skin | Lung | Spleen | Red blood cells | Brain | Fat |
| 0 | 5 |  |  |  |  |  |  |  |  |  |  |  |  |
| 0.5 | 0.1088 | 1.8954 | 0.44894 | 0.0462 | 0.1368 | 0.68198 | 0.4026 | 0.0124 | 0.1157 | 0.09079 | 0.6248 | 0.0094 | 0.0124 |
| 1 | 0.05576 | 0.3978 | 0.29366 | 0.0378 | 0.1102 | 0.60268 | 0.5368 | 0.0186 | 0.338 | 0.03017 | 1.42 | 0.0094 | 0.0186 |
| 4 | 0.02312 | 0.297 | 0.2344 | 0.0518 | 0.1368 | 0.65758 | 0.4026 | 0.0155 | 0.1586 | 0.00595 | 1.8744 | 0.0141 | 0.0155 |
| 7 | 0.02312 | 0.1728 | 0.13146 | 0.0462 | 0.057 | 0.31598 | 0.4026 | 0.0186 | 0.0364 |  | 1.9312 | 0.0188 | 0.0186 |
| 14 | 0.01632 | 0.0738 | 0.07906 | 0.0364 | 0.057 | 0.2806 | 0.2684 | 0.0124 | 0.0624 |  | 2.0732 | 0.0188 | 0.0124 |
| 28 | 0.02312 | 0.1152 | 0.06134 | 0.0574 | 0.0722 | 0.13176 | 0.671 | 0.0093 | 0.0208 |  | 1.9596 | 0.0188 | 0.0093 |

Iron adequate diet mice

| Time  (Days) | Fe59 amount in tissues (nmol) | | | | | | | | | | | | |
| --- | --- | --- | --- | --- | --- | --- | --- | --- | --- | --- | --- | --- | --- |
|  | Plasma | Bone | Gut | Heart | Kidney | Liver | Muscle | Skin | Lung | Spleen | Red blood cells | Brain | Fat |
| 0 | 5 |  |  |  |  |  |  |  |  |  |  |  |  |
| 0.5 | 0.11832 | 3.4866 | 0.4514 | 0.0728 | 0.19 | 1.03578 | 0.8052 | 0.5955 | 0.1014 | 0.05817 | 0.6106 | 0.0141 | 0.0124 |
| 1 | 0.0204 | 1.296 | 0.366 | 0.0364 | 0.1178 | 0.86132 | 0.4026 | 0.397 | 0.1521 | 0.08071 | 1.2283 | 0.0094 | 0.0186 |
| 4 | 0.01496 | 0.6858 | 0.1952 | 0.056 | 0.133 | 0.81008 | 0.4026 | 0.5558 | 0.0637 | 0.04333 | 1.5762 | 0.0141 | 0.0155 |
| 7 | 0.0068 | 0.5076 | 0.061 | 0.056 | 0.0646 | 0.66734 | 1.0736 | 0.5955 | 0.0429 | 0.04074 | 1.2638 | 0.0094 | 0.0341 |
| 14 | 0.01768 | 0.4518 | 0.0488 | 0.0518 | 0.0646 | 0.52704 | 0.1342 | 0.7146 | 0.0429 | 0.02786 | 1.2496 | 0.0141 | 0.0155 |
| 28 | 0.02176 | 0.2232 | 0.0366 | 0.0322 | 0.0684 | 0.31476 | 0.2684 | 0.2779 | 0.039 | 0.03066 | 1.1644 | 0.0094 | 0.0031 |

Iron loaded diet mice

| Time  (Days) | Fe59 amount in tissues (nmol) | | | | | | | | | | | | |
| --- | --- | --- | --- | --- | --- | --- | --- | --- | --- | --- | --- | --- | --- |
|  | Plasma | Bone | Gut | Heart | Kidney | Liver | Muscle | Skin | Lung | Spleen | Red blood cells | Brain | Fat |
| 0 | 5 |  |  |  |  |  |  |  |  |  |  |  |  |
| 0.5 | 0.1156 | 1.9116 | 0.28426 | 0.0938 | 0.285 | 0.93818 | 0.5368 | 0.397 | 0.1833 | 0.03682 | 0.1704 | 0.0047 | 0.0124 |
| 1 | 0.03536 | 1.2348 | 0.47214 | 0.1134 | 0.646 | 2.84504 | 1.0736 | 0.794 | 0.1742 | 0.06902 | 0.3692 | 0.0094 | 0.0279 |
| 4 | 0.00816 | 0.4212 | 0.20374 | 0.0896 | 0.38 | 1.8361 | 0.5368 | 0.3176 | 0.1898 | 0.04431 | 0.568 | 0.0047 | 0.0186 |
| 7 | 0.00544 | 0.3438 | 0.10126 | 0.1036 | 0.1786 | 1.0248 | 0.5368 | 0.397 | 0.3965 | 0.04963 | 0.852 | 0.0094 | 0.0217 |
| 14 | 0.00952 | 0.5472 | 0.09394 | 0.042 | 0.1558 | 1.0919 | 0.5368 | 0.3573 | 0.1638 | 0.04424 | 1.3064 | 0.0141 | 0.0217 |
| 28 | 0.01768 | 0.3168 | 0.07686 | 0.0378 | 0.133 | 1.09556 | 0.2684 | 0.3573 | 0.104 | 0.06496 | 0.9088 | 0.0094 | 0.0062 |

Rats

| Time  (h) | Serum  (μg/dL) |  |  | Time  (h) | Tissues (mg/kg) | | | | | |
| --- | --- | --- | --- | --- | --- | --- | --- | --- | --- | --- |
|  |  |  |  |  | Bone | Heart | Kidney | Liver | Muscle | Spleen |
| 1 | 1147.87 |  |  | 72 | 42.40 | 64.54 | 36.65 | 341.36 | 11.83 | 449.76 |
| 2 | 979.73 |  |  | 192 | 38.99 | 51.70 | 25.70 | 236.92 | 11.27 | 461.72 |
| 4 | 625.86 |  |  | 360 | 15.27 | 40.42 | 19.92 | 84.11 | 7.70 | 385.17 |
| 6 | 839.33 |  |  |  |  |  |  |  |  |  |
| 8 | 660.93 |  |  |  |  |  |  |  |  |  |
| 12 | 526.43 |  |  |  |  |  |  |  |  |  |
| 24 | 438.72 |  |  |  |  |  |  |  |  |  |
| 48 | 111.29 |  |  |  |  |  |  |  |  |  |

Humans

| Time  (Days) | Serum iron concentration (μg/mL) | | | |
| --- | --- | --- | --- | --- |
|  | 100 mg | 500 mg | 800 mg | 1000 mg |
| 0.25 | 36.3 | 153.23 | 287.72 | 322.87 |
| 0.5 | 36.1 | 150 | 292.61 | . |
| 0.75 | 34.73 | 148.39 | 272.89 | 308.37 |
| 1 | 33.17 | 141.94 | 287.63 | 318.46 |
| 1.5 | 31.69 | 140.32 | 292.47 | 320.78 |
| 2 | 31.27 | 133.87 | 262.90 | 308.09 |
| 3 | 26.97 | 127.42 | 285.71 | 297.72 |
| 4 | 25.50 | 122.58 | 239.67 | 288.99 |
| 6 | 20.92 | 101.61 | 218.10 | 261.61 |
| 8 | 17.97 | 88.71 | 185.03 | 240.79 |
| 12 | 13.66 | 69.35 | 141.87 | 215.71 |
| 16 | 12.59 | 51.61 | 124.93 | 175.79 |
| 24 | 8.82 | 30.65 | 79.59 | 135.21 |
| 36 | 7.75 | 12.90 | 29.87 | 78.74 |
| 48 | 7 | 8.06 | 11.57 | 54.64 |
| 60 |  | 7 | 8.74 | 22.10 |
| 72 |  |  | 7 | 7 |
